# Downregulation of a UDP-Arabinomutase Gene in Switchgrass (*Panicum virgatum* L.) Results in Increased Cell Wall Lignin While Reducing Arabinose-Glycans

**DOI:** 10.3389/fpls.2016.01580

**Published:** 2016-10-27

**Authors:** Jonathan D. Willis, James A. Smith, Mitra Mazarei, Ji-Yi Zhang, Geoffrey B. Turner, Stephen R. Decker, Robert W. Sykes, Charleson R. Poovaiah, Holly L. Baxter, David G. J. Mann, Mark F. Davis, Michael K. Udvardi, Maria J. Peña, Jason Backe, Maor Bar-Peled, C. N. Stewart

**Affiliations:** ^1^Department of Plant Sciences, University of Tennessee, KnoxvilleTN, USA; ^2^BioEnergy Science Center, Oak Ridge National Laboratory, Oak RidgeTN, USA; ^3^Complex Carbohydrate Research Center, University of Georgia, AthensGA, USA; ^4^The Samuel Roberts Noble Foundation, ArdmoreOK, USA; ^5^The National Renewable Energy Laboratory, GoldenCO, USA; ^6^Plant Biology, University of Georgia, AthensGA, USA

**Keywords:** switchgrass, hemicellulose arabinoxylan, UDP-arabinopyranose mutase/reversible glycosylated polypeptide, biofuel, recalcitrance

## Abstract

**Background:** Switchgrass (*Panicum virgatum* L.) is a C_4_ perennial prairie grass and a dedicated feedstock for lignocellulosic biofuels. Saccharification and biofuel yields are inhibited by the plant cell wall’s natural recalcitrance against enzymatic degradation. Plant hemicellulose polysaccharides such as arabinoxylans structurally support and cross-link other cell wall polymers. Grasses predominately have Type II cell walls that are abundant in arabinoxylan, which comprise nearly 25% of aboveground biomass. A primary component of arabinoxylan synthesis is uridine diphosphate (UDP) linked to arabinofuranose (Ara*f*). A family of UDP-arabinopyranose mutase (UAM)/reversible glycosylated polypeptides catalyze the interconversion between UDP-arabinopyranose (UDP-Ara*p*) and UDP-Ara*f*.

**Results:** The expression of a switchgrass arabinoxylan biosynthesis pathway gene, *PvUAM1*, was decreased via RNAi to investigate its role in cell wall recalcitrance in the feedstock. *PvUAM1* encodes a switchgrass homolog of UDP-arabinose mutase, which converts UDP-Ara*p* to UDP-Ara*f*. Southern blot analysis revealed each transgenic line contained between one to at least seven T-DNA insertions, resulting in some cases, a 95% reduction of native *PvUAM1* transcript in stem internodes. Transgenic plants had increased pigmentation in vascular tissues at nodes, but were otherwise similar in morphology to the non-transgenic control. Cell wall-associated arabinose was decreased in leaves and stems by over 50%, but there was an increase in cellulose. In addition, there was a commensurate change in arabinose side chain extension. Cell wall lignin composition was altered with a concurrent increase in lignin content and transcript abundance of lignin biosynthetic genes in mature tillers. Enzymatic saccharification efficiency was unchanged in the transgenic plants relative to the control.

**Conclusion:** Plants with attenuated *PvUAM1* transcript had increased cellulose and lignin in cell walls. A decrease in cell wall-associated arabinose was expected, which was likely caused by fewer Ara*f* residues in the arabinoxylan. The decrease in arabinoxylan may cause a compensation response to maintain cell wall integrity by increasing cellulose and lignin biosynthesis. In cases in which increased lignin is desired, e.g., feedstocks for carbon fiber production, downregulated *UAM1* coupled with altered expression of other arabinoxylan biosynthesis genes might result in even higher production of lignin in biomass.

## Introduction

Switchgrass (*Panicum virgatum*) is a perennial grass species that is considered to be a lignocellulosic bioenergy feedstock with great potential, owing to its wide adaptations to various geographies and temperate climates. Recalcitrance, which is the inherent resistance of cell wall polysaccharides to be digested into fermentable sugars, is a sizeable economic barrier to lignocellulosic biofuel production. At the center of recalcitrance is the heterogeneous composition of plant cell walls, which are made of three main types of polymers: cellulose, lignin, and hemicellulose (Dixon, 2013). Feedstock genomics and biotechnology have enabled a better understanding of cell wall recalcitrance, including that for switchgrass (Casler et al., 2011; Chen et al., 2016). Relatively few studies exist in which hemicellulose has been manipulated were carried out to determine its role in cell wall recalcitrance in biofuel crops (Vega-Sanchez and Ronald, 2010).

In plants, hemicelluloses are comprised of non-cellulose cell wall polysaccharides, and share a sugar backbone composed of 1,4-linked β-D-glycoses and include xyloglucan mixed-linkage glucan, xylan, and glucomannan. Xylan itself constitutes a sub-grouping of polysaccharides whose members are distinguished from one another by the types of oligosaccharide side chains linked to the 1,4-linked β-D-xylopyranose (Xyl*p*) backbone (York and O’Neill, 2008; Scheller and Ulvskov, 2010; Rennie and Scheller, 2014). The backbone of xylans isolated from grasses for example, is decorated by a large number of L-arabinose residues (found only in furanose form, Ara*f*), and hence referred to as arabinoxylans. The Ara*f* residues are attached to the Xyl*p* residues in the backbone predominately at *O*-3 but occasionally at *O*-2. Some of these Ara*f* residues are linked at *O*-2 with an additional α-L-Ara*f* or a β-D-Xyl*p* residue. Grass xylans also contain small amounts of glucuronic acid (GlcA) and methylated glucuronic acid (MeGlcA) side chains at *O*-2 (Ebringerova and Heinze, 2000). Xylan may also contain non-carbohydrate modification of *O*-acetyl esters and methyl etherified sugars, as well as feruloyl, and *p*-coumaroyl moieties (Faik, 2010; Bar-Peled and O’Neill, 2011). For example, the aromatic residues (feruloyl and *p*-coumaroyl) can be ester-linked to *O*-5 of terminal or substituted arabinose residues of xylan, whereas the acetate can be attached at *O*-2, *O*-3 or both to xylose in the backbone. In grass species, ferulic acid is ester-linked to the C5 hydroxyl of Ara*f* in arabinoxylan and in ether linkages of lignin monomers (Scalbert et al., 1985; Hartley and Ford, 1989). Although the role of feruloylation is not well understood, an increase in ferulic acid modification of arabinoxylan has been associated with cells that have stopped elongating (Carpita, 1986). Feruloylation has been hypothesized to prime polymerization of lignin thereby interconnecting a network of xylan and lignin (Iiyama et al., 1994; de and Buanafina, 2009). Additionally, adjacent arabinoxylan chains decorated with ferulic acid can dimerize through oxidative coupling, which may condense wall polymers into a tightly packed matrix enhancing the walls stability and resistance to degradation (Hatfield et al., 1999). Disruption of these ether linkages between arabinoxylan and lignin is an inviting target for improving cell wall degradation.

The diversity in xylan structures is known, but the functional role for such diversity is largely unknown. For example, it is not understood why xylan chemotypes differ among tissues in the same plants. It was proposed that xylan interacts with cellulose and lignin, which serves to strengthen cell walls (Scheller and Ulvskov, 2010). Arabinoxylans comprise over 25% of the mass of grass cell walls (Faik, 2010; Konishi et al., 2011). The formation of arabinoxylan requires the building blocks uridine diphosphate (UDP)-xylose and UDP-arabinofuranose (UDP-Ara*f*). UDP-arabinopyranose mutase (UAM) converts UDP-arabinose (pyranose-form, Ara*p*) to UDP-Ara*f* (Konishi et al., 2007, 2010). UAM orthologs are found in some microalgae and land plants in which they comprise a small gene family (Kotani et al., 2013). Interestingly, UAM can also reversibly glycosylate itself in the presence of UDP-sugars, such as UDP-glucose, UDP-galactose, and UDP-xylose [hence the name RGP (reversible glycosylated polypeptide); Dhugga et al., 1991; Konishi et al., 2007, 2010; Rautengarten et al., 2011]. The role of UAM as an RGP is not well understood in the context of cell wall and glycan formation. It was hypothesized that the RGP function of UAM may regulate the internal balance of UDP-sugars in the cell or compete for the formation of UDP-Ara*f*, and this hypothesis has been explored in various taxa including *Arabidopsis*, algae, *Brachypodium*, and rice (Konishi et al., 2011; Rautengarten et al., 2011; Kotani et al., 2013; Rancour et al., 2015). However, UAM’s potential role in recalcitrance has never been examined nor manipulated in any bioenergy feedstock.

In this study, it was hypothesized that manipulation of the level of UDP-Ara*f* in cells would alter the amount of arabinoxylan in switchgrass cell walls, and potentially alter feedstock recalcitrance in aboveground biomass. In this study, a switchgrass *UAM1* homolog (*PvUAM1*) was downregulated in independent transgenic lines of switchgrass, wherein cell wall composition, saccharification, and plant growth were analyzed.

## Materials and Methods

### *PvUAM1* Gene Isolation and RNAi Construct

The amino acid sequence of switchgrass UAM protein was compared with UAM orthologs from eudicots and monocots: SiUAM1 (XP004982467.1), ZmUAM1 (NP001105598.1), SbUAM1(XP002464260.1),OsUAM1(XP006650286.1),BdUAM1 (XP003562308.1), TaUAM1 (CAA77237.1), SlUAM1 (NP001234554.1), VvUAM1 (XP002263490.1), BdUAM1 (XP003569874.1), GmUAM1 (XP003552602.1), BrUAM1 (XP009117866.1), AtUAM3 (AAM65020.1), AtUAM1 (AT3G02230.1), PtUAM1 (Potri.004G117800.1), MtUAM1 (Medtr5g046030.1), OsUAM1 (Q8H8T0.1), OsUAM3 (Q6Z4G3.1), OsUAM2 (Q7FAY6.10), AtUAM2 (NP197069.1), EgUAM1 (AGE46030.1), PdUAM1 (XP008811806.1). The sequences among UAM proteins were compared using alignment using the MUSCLE program^1^ and alignments were curated by Gblocks using the Phyologeny.fr software program^2^ (Anisimova and Gascuel, 2006; Dereeper et al., 2008). The neighbor-joining tree was generated using the MEGA 7.0 program (Tamura et al., 2013). The switchgrass *PvUAM1. PvUAM2. PvUAM3*, gene sequences were identified by BLASTN analysis of the switchgrass genome^3^ using the monocot UAM sequences from maize (GI: 542592), foxtail millet (GI: 101771463), and sorghum (GI: 8062976). The nucleotide coding sequence of the *PvUAM1* open reading frame was identified and a 193 bp target sequence was used to generate the RNAi plasmid construct (**Supplementary Figure S1**). The target sequence was amplified by PCR and was cloned into the pCR8 entry vector and then Gateway^®^ sub-cloned into the pANIC-8A plant expression vector (Mann et al., 2012b) to yield the pANIC-8A-PvUAM1 construct (**Supplementary Figure S2A**).

### Transgenic Plant Production and Growth Analysis

Inflorescences of the “Alamo” switchgrass “ST1” genotype was used to generate Type II embryogenic callus (Burris et al., 2009). *Agrobacterium tumefaciens* strain EHA105 harboring the pANIC-8A-PvUAM1 vector was used for transformation. Transformed calli were grown on solidified LP9 growth medium (Burris et al., 2009), supplemented with 400 mg/L Timentin (ticarcillin disodium and clavulanate potassium) and 40 mg/L hygromycin for approximately 2 months at 25°C in the dark. Subsequently the transgenic calli were transferred to regeneration medium as described by Li and Qu (2011) and was supplemented with 250 mg/L cefotaxime to stimulate regeneration (Danilova and Dolgikh, 2004). The T-DNA region of pANIC-8A-PvUAM1 plasmid also contains a cassette that constitutively expresses an orange fluorescence protein (OFP) reporter from the hard coral *Porites porites* (pporRFP) that is brightly fluorescent in transgenic plants (Mann et al., 2012a). Epifluorescence microscope having a 535/30 nm excitation filter and 600/50 nm emissions filter was used to track OFP fluorescence during transgenic callus development and to identify individual putative transgenic lines during growth on agar-plate. Regenerated transgenic plants were rooted and acclimated according to Burris et al. (2009).

T0 transgenic and non-transgenic ST1 control plants were grown in growth chambers under 16 h light/8 h dark cycles at 25°C until moved to a greenhouse with approximately the same conditions. Fertilizer (0.02% solution of Peter’s soluble 20-20-20) was applied twice per month and plants were irrigated as needed. For growth analysis, each transgenic event and the non-transgenic control was vegetatively divided into three clonal replicates. The non-transgenic (ST1) control was derived from callus that was treated identically as the transgenics with the exception of not being exposed to *A. tumefaciens* nor hygromycin each replicate, starting from a single-tiller, was grown in its own a 4 L pot that was randomly sited in the greenhouse. Plants were grown until the reproductive (R1) developmental stage as defined by Hardin et al. (2013) and tiller and panicle numbers were counted. The five tallest tillers for each replicate were used to estimate total plant height. The stem width at 10 cm from the potting surface of each of these tillers was measured with a digital caliper. Tillers were harvested and green fresh weight was recorded. Harvested tillers were placed into a drying oven at 42°C for 5 days and dry weight was subsequently recorded. Hand sectioning was performed on fresh tillers and nodal sections to assess vascular phenotypes under a dissecting microscope and to depict deposition of pigment.

### Southern Blot Analysis for T-DNA Copy Number

Approximately 100 mg of young (1-week-old from recently cut-back plants) fresh leaf tissue per plant was used to extract DNA (Freeling and Walbot, 1994). DNA quality was assessed using gel electrophoresis and quantified using a Nanodrop spectrofluorometer (Thermo Fisher, Wilmington, DE, USA). Twenty micrograms of DNA from each sample was digested with *Nco*I, which cuts once within the T-DNA. Digested DNA from transgenic plants and the control, was separated on a Tris-acetate-EDTA (TAE)-agarose gel and transferred to a nylon membrane (Amersham Hybond^TM^ + GE Healthcare, Pittsburgh, PA, USA). Blots were pre-hybridized with pre-hybridized with digoxigenin (DIG) easy hyb (Roche DIG kit, Nutley, NJ, USA) solution at 42°C. The blots were then hybridized with the hygromycin DIG-PCR probe, washed, and probe was detected after the membrane was exposed to x-ray film according to manufacturer’s instructions (Roche). The DNA probe (972 bp) used to detect the number of hygromycin (*hph*) gene cassette in DNA from transgene lines was amplified by PCR and labeled with digoxigenin (Roche).

### RNA Extraction, qRT-PCR Analysis of UAMs and Lignin Biosynthetic Gene Transcripts

Quantitative RT-PCR was performed to estimate transcript abundance of *PvUAM* and lignin biosynthetic genes in transgenic *PvUAM1*-RNAi and non-transgenic plants. Total RNA was isolated from triplicate R1 tiller stem internodes and leaf cuttings using TRI Reagent^®^ following manufacturer’s instructions (Sigma-Aldrich, St. Louis, MO, USA). Purified RNA was treated with DNase-1 (Promega, Madison, WI, USA) and 3 μg treated RNA was used to generate cDNA using oligo-dT and Superscript III according to manufacturer’s instructions (Life Technologies, Carlsbad, CA, USA). qRT-PCR analysis was performed with Power SYBR Green PCR master mix according to manufacturer’s protocols (Life Technologies) for optimization of annealing temperature, primer concentration, and cDNA concentration. Primers used for transcript analysis of *PvUAM* are listed in **Supplementary Table S1** and for lignin biosynthetic genes in **Supplementary Table S2**. The optimized qRT-PCR protocol utilized a dilution of cDNA 1:100 with thermal cycling at 95°C for 3 min, and 40 cycle repeats of (95°C for 10 s and 60.0°C for 30 s). The relative levels of transcripts were normalized to switchgrass ubiquitin 1 (*PvUbi1*) as a reference gene (Shen et al., 2009) using primer set: PvUBi1_F 5′-CAGCGAGGGCTCAATAATTCCA-3′ and PvUbi1_R 5′-TCTGGCGGACTACAATATCCA-3′ (Xu et al., 2011). All experiments were carried out in triplicate technical replicates. The differential Ct method was used to measure transcript abundance after normalization to *PvUbi1* (Schmittgen and Livak, 2008).

### Glycosyl Residue Composition and Gas Liquid Chromatography Analysis

Tillers at the R1 developmental stage were collected from a single plant grown in a greenhouse for approximately 6–8 weeks. The tillers were cut and divided into stem and leaf sections. A sample section was weighed, ground in liquid nitrogen, and washed as previously reported (Martinez et al., 2012) with slight modifications. Each 1 g sample was suspended in 10 mL 80% EtOH, vortexed for 2 min, then centrifuged (6,000 × *g* 5 min, 25°C). The supernatant was removed, and the resulting cell pellet was washed two times each with 10 mL 95% EtOH, and then with 10 mL 100% EtOH. The cell pellet was resuspended in 10 mL chloroform:MeOH 1:1 (v/v) and mixed by tilting for 1 h. Each cell pellet residue sample was filtered through Whatman # 15 filter paper over vacuum and rinsed with acetone. Once dry, the alcohol-insoluble residue (AIR) samples were weighed and passed through a 0.5 mm mesh. AIR sample (10 mg) was suspended in 1 mL buffer (0.1 M sodium acetate, 0.01% Thimerosal, pH 5.0) and treated with an amylase mixture; Spirizyme Excel (1.2 μL) and Liquozyme SC DS^®^ (6 μL) (Novozymes, Bagsværd, Denmark; # NAPFM084 and AUP61163, respectively), as described by Decker et al. (2012). Starch digestion was carried out at 55°C overnight and subsequently the slurry sample was filtered through double filter layers (50 μm nylon mesh on top of Whatman Grade GF/A filter), and washed with buffer. The polysaccharides in each 1 mg AIR sample were hydrolyzed with 2N trifluoroacetic acid and the free monosaccharides were converted to their alditol acetate derivatives as previously described (York et al., 1986). All samples (including sugar standards) were supplemented with 50 μL 5 mM inositol as an internal standard. Alditol acetate sample or standard (1 μL) was separated on a Restek RTx-2330 fused silica column (0.25 mm I.D. × 30 m, 0.2 μm film thickness) using an Agilent 7890A GC equipped with a flame ionizing detector (Agilent, Santa Clara, CA, USA). Relative molar percent content was calculated from the areas of sugar peaks identified by standard retention times and normalized to sample mass and internal standard.

### Isolation and Fractionation of Wall Polysaccharides and Oligosaccharides

AIR sample (250 mg) was de-starched as above in 25 mL buffer treated with 30 μL Spirizyme Excel and 150 μL Liquozyme SC DS at 55°C overnight, filtered and washed with buffer. To remove loosely bound pectin, the amylase-insoluble residue was resuspended in 25 mL oxalate solvent (0.5% ammonium oxalate, 0.01% Thimerosal, pH 5.0) and shaken overnight at room temperature. The slurry was filtered through double filter layers, washed with oxalate, and the insoluble residue was reserved. Each oxalate-treated residue was resuspended in 25 mL 1 M base solution (1 M KOH, 1% NaBH_4_) and shaken overnight at room temperature. The slurry was filtered through double filter layers. The filtrate that contained soluble polysaccharide was reserved and the insoluble residue slurry on top of double layer filter was wash with 1 M KOH and insoluble pellet was reserved. The filtrate and wash 1 M KOH-solubilized wall polymers were combined, supplemented with a drop of octanol as antifoam, neutralized to pH 7 with glacial acetic acid and later dialyzed (3500 molecular weight cut off) against deionized water for 2–3 days. The insoluble residue after 1 M KOH treatment was resuspended in 25 mL 4 M base solution (4 M KOH, 1% NaBH_4_), shaken overnight at room temperature and filtered. The 4 M KOH soluble fraction was neutralized and dialyzed as described above. The dialyzed KOH fractions were centrifuged (11,000 × *g* for 30 min, 25°C), concentrated by Rotavapor, lyophilized, and used to generate oligosaccharides by enzymatic digestion. The 4 M KOH insoluble residue (enriched in cellulose) was stored at -20°C for analysis of cellulose.

### Preparation of Arabinoxylooligosaccharides and Nuclear Magnetic Resonance (NMR) Analysis

Between 5 and 20 mg of 1 M KOH soluble and dialyzed fraction (above) was dissolved in 1–5 mL of 50 mM ammonium formate, pH 5.0. One unit of endoxylanase (from *Trichoderma viride*, Megazyme, Wicklow, Ireland) was added and the solution incubated at 37°C for 24 h. Hydrolase activity was terminated by boiling for 10 min in a water bath and the sample was centrifuged at 3,600 × *g* for 15 min at room temperature. The supernatant was transferred to a tube and lyophilized. A portion of freeze-dried arabinoxylooligomers (1–2 mg) was dissolved in 0.5 mL deuterium oxide (99.9%; Cambridge Isotope Laboratories, Tewksbury, MA, USA) and supplemented with 1 μL acetone that was used as an internal chemical shift reference. One-dimensional and two-dimensional ^1^H NMR spectra were collected on a 600 MHz Varian Inova NMR spectrometer equipped with a 3-mm cold probe and a sample temperature of 25°C. Data were processed with MestReNova (Version 9.1; Mestrelab Research, Santiago de Compostela, Spain). All chemical shifts were measured relative to internal acetone (δ H 2.225).

### Cellulose Quantification

Thirty milligrams of 4 M KOH insoluble residue was weighed into a conical borosilicate tube with Teflon-lined screw cap, and 3 mL solvent (acetic acid/water/nitric acid, 8/2/1, v/v/v) was added (Updegraff, 1969). The sample was vortexed, heated in a boiling water bath for 30 min with occasional mixing, cooled to room temperature, and centrifuged (2,500 × *g* for 3 min). The pellet was re-suspended twice in 5 mL water, centrifuged, and the supernatant discarded. The enriched cellulose pellet was treated with 2.5 mL of 72% sulfuric acid (Updegraff, 1969) and incubated at room temperature for 1 h while mixing every 10 min by vortex. Samples were then transferred to a 15 mL Falcon tube and water added to 10 mL. Ten microliters of solution was transferred to a new borosilicate tube and diluted to 400 μL with water. One milliliter of ice-cold anthrone reagent [0.2 g anthrone in 100 mL concentrated sulfuric acid (95–98%)] was added, and the mixture was heated in a boiling water bath for 15 min. Following the anthrone reaction, the amount of sugars in the cellulosic polysaccharide fraction of nitric acid-treated unfractionated cellulose was determine by measuring absorbance at 620 nm with a DU 800 series spectrophotometer (Beckman Coulter, Brea, CA, USA) using glucose from Avicel as standard.

### Cell Wall Sugar Release and Lignin Content and Composition

Tillers were collected at the R1 developmental stage from greenhouse-grown plants and air dried for 3 weeks at room temperature before grinding to 1 mm (20 mesh) particle size. Sugar release efficiency was determined via National Renewable Energy Laboratory (NREL) high-throughput sugar release assays on extractive- and starch-free samples using glycosyl hydrolases according to NREL protocol (Selig et al., 2010; Decker et al., 2012). Glucose and xylose release was determined by colorimetric assays with total sugar release being the sum of glucose and xylose released. Lignin analysis was performed on the same samples described above. The lignin content and composition was determined by high-throughput pyrolysis molecular beam mass spectrometry (py-MBMS) on starch-free samples (Sykes et al., 2009) at the NREL (Golden, CO, USA). Additionally, *p*-hydroxyphenyl (H) lignin analysis was determined by thioacidolysis according to NREL protocol (Harman-Ware et al., 2016).

### Statistical Analysis

Statistical analysis was carried out with biological and technical replicates using SAS^®^ (Version 9.3; SAS Institute Inc., Cary, NC, USA) programming of mixed model ANOVA and least significant difference. This statistic analyses was performed on *PvUAM1* and *PvUAM1* homolog transcript abundance by qRT-PCR, growth analysis, cell wall-associated sugar content, NMR sugar side chain analysis, cellulose quantification, enzymatic sugar release, lignin content and composition, and lignin biosynthesis gene quantification by qRT-PCR. The standard error of the mean was calculated and displayed as error bars. *p*-Values of ≤0.05 were considered to be statistically significant.

## Results

### Identification of PvUAM Homologs

**FIGURE 1:**
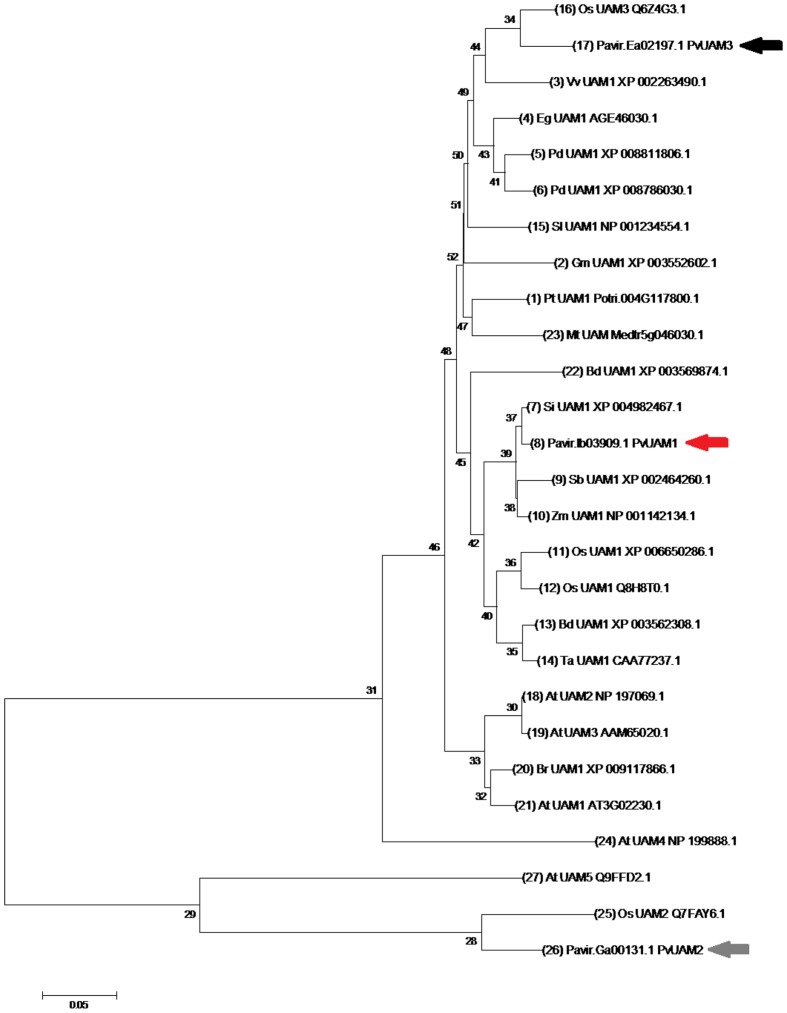
**Neighbor-joining cluster analysis of UAM amino acids.** The numerals on branches represent number of amino acid substitutions per site for known UAM protein sequences. The scale bar shows 0.05 amino acid substitutions per site. Switchgrass UAM homologs are indicated by arrows, PvUAM1 (Pavirv000Ib03909; red), PvUAM2 (Pavir.GA00131.1; gray), PvUAM3 (Pavir.EA02197.1; black). Tree generated using the MEGA 7.0 program (Tamura et al., 2013) of UAM amino acid sequence alignments using Gblocks at the phylogeny.fr website (http://phylogeny.lirmm.fr). Analysis using 1000 bootstrap replicates was performed.

Orthologous of functional UDP-arabinomutase (UAM1) amino acid sequences from monocot and eudicot plant species were used to identify the switchgrass PvUAM1 sequence (**Figure 1**). The UAM1 has additional function or reversibly glycosylated protein (RGP1), hence forward be named UAM1/RGP1 or UAM1. PvUAM1 has 93 and 86% amino acid sequence similarity to the rice UAM1 and Arabidopsis UAM1/RGP1, respectively. Sequence relationships of UAM proteins from diverse plant species grouped into a central monocot cluster and a split eudicot grouping. PvUAM1 belongs to the monocot group. In addition to UAM1, two other UAM-homologs are known. PvUAM1 is 48% similar to PvUAM2 whereas PvUAM1 has 86% amino acid sequence similarity to PvUAM3.

### Molecular and Phenotypic Characterization of PvUAM-RNAi Transgenic Plants

**FIGURE 2:**
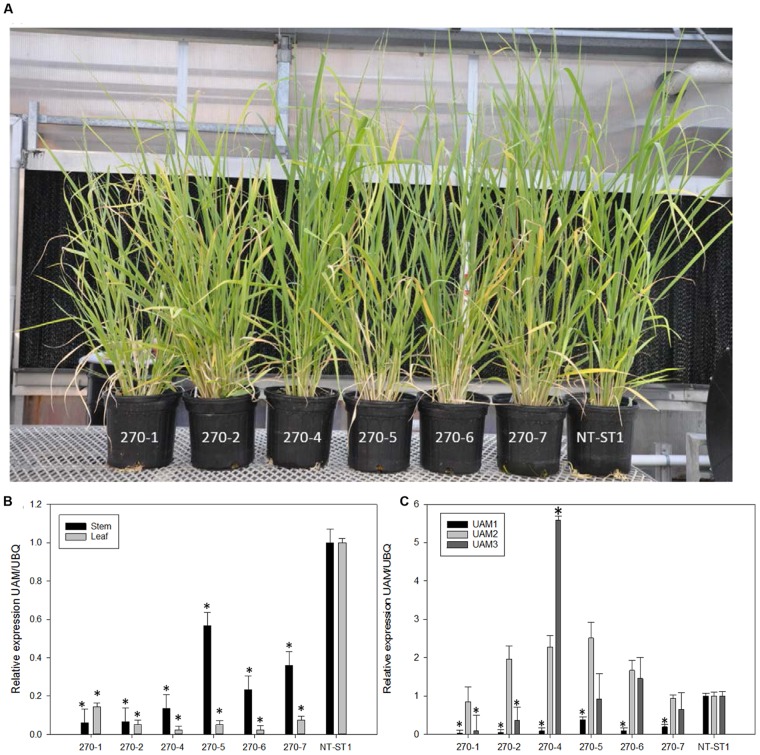
**(A)** Representative downregulated *PvUAM1* transgenic and non-transgenic (NT-ST1) switchgrass lines. **(B)** Relative expression of *PvUAM1* in leaf and stem tissues of transgenic and non-transgenic lines. **(C)** Relative expression of *PvUAM1. PvUAM2*, and *PvUAM3* in stem tissue of transgenic and non-transgenic lines. Relative expression analysis were determined by qRT-PCR and normalized to switchgrass ubiquitin 1 (*PvUbi1*). Bars represent mean values of three replicate stem internode or leaves ± standard error. Asterisks indicate significant differences from non-transgenic control plants at *p* ≤ 0.05 as tested by least significant difference (LSD) method.

To study the role of switchgrass PvUAM1 in hemicellulose metabolic pathways RNAi-transgenic switchgrass plants were generated. Seven independent transgenic events regenerated from transformed callus were analyzed (**Figure 2A**). Southern blot analysis showed that each transgenic line carried at least one and up to seven T-DNA inserts (**Supplementary Figure S2B**). One transgenic line (270-3) did not survive and was removed from subsequent analysis. The *PvUAM1* transcript abundance was less than that of the control in each of six remaining transgenic lines in both stems and leaves. For example, *PvUAM1* transcript level in stems and leaves of the RNAi plant lines, decreased by 67–95% and to 77–98% relative to the non-transgenic control, respectively (**Figure 2B**). Gene expression analysis of *PvUAM* homologs (*PvUAM1. PvUAM2*, and *PvUAM3*) was performed on stem internode sections (**Figure 2C**). *PvUAM2* expression amongst all transgenic lines was not significantly different than the control. However, and although unintended, the *PvUAM1* RNAi target sequence was similar enough to cause significant downregulation in both *PvUAM1* and *PvUAM3* homologs for lines 270-1 and 270-2. *PvUAM3* transcript was significantly reduced in lines 270-1 and 270-2 by 90 and 74% relative to the control, respectively. An opposite effect was observed for 270-4 in which *PvUAM3* was found to be upregulated 4.6-fold over the control. *PvUAM3* transcript abundance was unchanged in 270-5, 270-6, and 270-7 compared to the non-transgenic control. Interestingly, we found no apparent correlation between number of T-DNA insert and the reduced transcript abundance of UAM1 in these transgenes.

**FIGURE 3:**
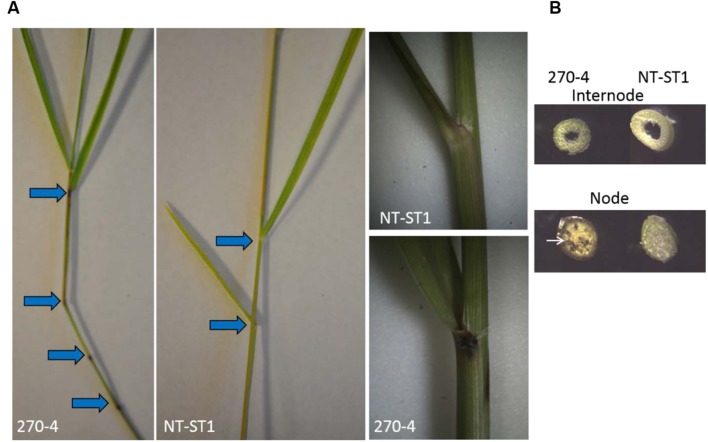
**Stem node phenotype from fresh E3 (elongation growth stage) tillers in downregulated *PvUAM1* transgenic switchgrass.** **(A)** Comparison of transgenic *PvUAM1* (270-4) and non-transgenic (NT-ST1) nodes and internodes. Arrows indicate nodes. **(B)** Cross-sections of vascular bundles at nodes and internodes of transgenic and non-transgenic plants. Arrow indicates darkened vascular bundle.

There were several instances of altered plant growth among the transgenic switchgrass lines (**Table 1**). Transgenic plant lines 270-1, 270-2, 270-5, and 270-7 had equivalent number of tillers compared to the control whereas lines 270-4 and 270-6 had significantly more tillers per plant. Plant lines 270-1, 270-2, and 270-6 were shorter, whereas 270-4 and 270-5 were equivalent to control plants. Line 270-7 was taller when compared with control line. Tiller stem width was significantly reduced up to 22% in lines 270-1, 270-2, and 270-6, but was increased in 270-7 by up to 6%, whereas the remainder of the lines had unchanged stem width from the control. Fresh weight was significantly increased from the control by up to 102% in lines 270-2, 270-4, 270-5, and 270-7, whereas lines 270-1 and 270-6 were equivalent to control. Dry biomass results were similar to that of fresh weight except line 270-6 was also higher than control. Panicle number was significantly increased in all transgenic lines. In addition to the above-mentioned phenotypic differences between PvUAM-RNAi lines and control we interestingly found that line 270-1, 270-2, and 270-4 appeared to have an increased level of red pigment in the stem nodes when compared with the non-transgenic plants (**Figure 3A**). Cross sectioning of fresh stem nodes and internodes at the E3 (elongation) developmental stage on plant line 270-4 showed a dark pigmentation that was deposited in the vascular bundles and outer tissue of the nodes (**Figure 3B**).

**Table 1:** Growth of downregulated *PvUAM1* transgenic and non-transgenic (NT-ST1) switchgrass lines.

Line	Tiller number	LSD	Tiller height (mm)	LSD	Stem width (mm)	LSD	Panicle number	LSD	Fresh weight (g)	LSD	Dry weight (g)	LSD
270-1	32.0 ± 4.9	AB	873.67 ± 17.9	D	3.61 ± 0.12	C	3.7 ± 0.8	ABC	87.70 ± 16.24	BC	22.86 ± 4.37	BC
270-2	29.0 ± 4.9	AB	923.9 ± 17.9	D	3.68 ± 0.12	C	5.3 ± 0.8	AB	165.24 ± 16.24	A	40.21 ± 4.37	A
270-4	42.3 ± 4.9	A	1089.1 ± 17.9	BC	4.12 ± 0.12	AB	5.0 ± 0.8	AB	170.35 ± 16.24	A	39.72 ± 4.37	A
270-5	33.7 ± 4.9	AB	1087.2 ± 17.9	BC	4.37 ± 0.12	A	3.3 ± 0.8	BC	135.81 ± 16.24	AB	35.64 ± 4.37	AB
270-6	40.0 ± 4.9	A	1039.9 ± 17.9	C	3.88 ± 0.12	BC	5.7 ± 0.8	A	128.15 ± 16.24	ABC	34.15 ± 4.37	AB
270-7	36.0 ± 4.9	AB	1185.9 ± 17.9	A	4.36 ± 0.12	A	4.3 ± 0.8	ABC	174.21 ± 16.24	A	41.23 ± 4.4	A
NT-ST1	22.0 ± 4.9	B	1118.3 ± 17.9	B	3.85 ± 0.12	BC	2.7 ± 0.8	C	86.34 ± 16.24	C	18.62 ± 4.37	C


*Tiller number refers to the mean tally of all tillers per pot present at time of collection. Tiller height refers to the mean value of the five tallest tillers per replicate pot. Stem width of the five tallest tillers per replicate pot was measured at the potting level and averaged. Panicle number refers to the mean value of panicles present at time of collection per pot. Fresh weight refers to the mean value of fresh total biomass collected per pot. Dry weight refers to the mean value of total biomass collected and then dried for 5 days at 42°C per pot. Error bars represent standard error of the mean of three whole plant replicates. Values that share the same letter are not significantly different as calculated by LSD (*p* ≤ 0.05).*

### The Wall of PvUAM-RNAi Transgenic Plants Have Reduced Arabinose

**FIGURE 4:**
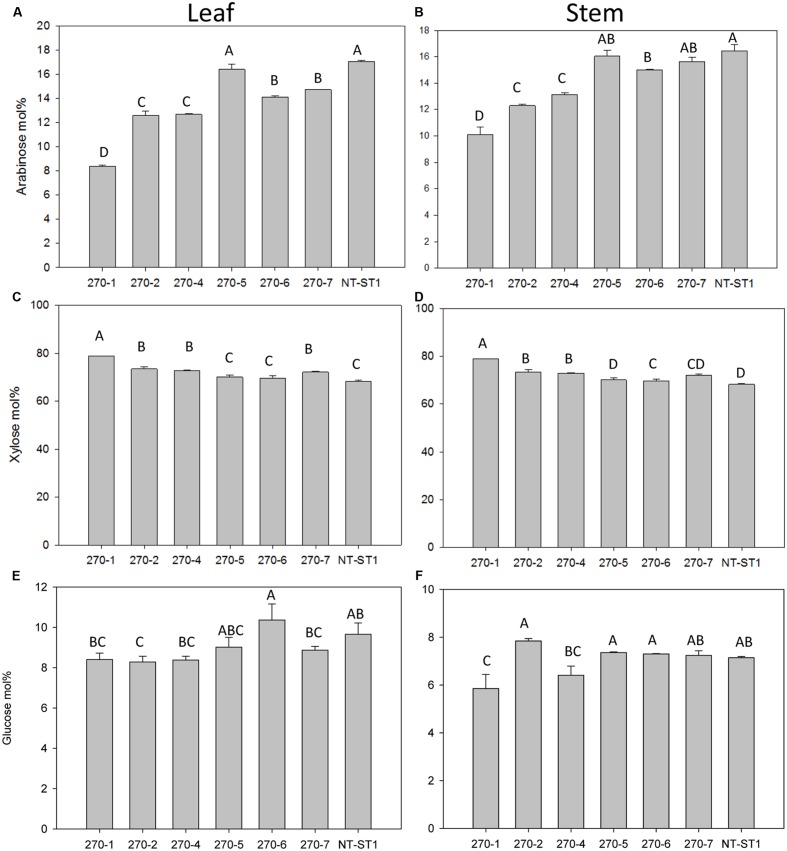
**Arabinose **(A,B)**, xylose **(C,D)**, and glucose **(E,F)** content in leaf **(A,C,E)** and stem **(B,D,F)** of transgenic and non-transgenic (NT-ST1) lines as determined by gas chromatography.** Samples were normalized to internal standard (inositol) with mol% representing the % of total cell wall-associated sugars measured. Bars represent mean values of three biological replicates ± standard error. Bars represented by same letters are not significantly different as calculated by LSD (*p* ≤ 0.05).

Following phenotypic analyses of PvUAM-RNAi transgenic plants we determined the sugar composition of polysaccharides in the cell walls of these lines. When compared with wall the control, the cell walls of leaves from transgenic PvUAM-RNAi lines had up to 51% decreased arabinose content (**Figure 4A**). Similarly the wall from transgenic stem showed up to 39% decreased arabinose content (**Figure 4B**). The highest reduction in arabinose was observed in leaf and stem of line 270-1, which also exhibited the greatest degree of *PvUAM1* knockdown.

**FIGURE 5:**
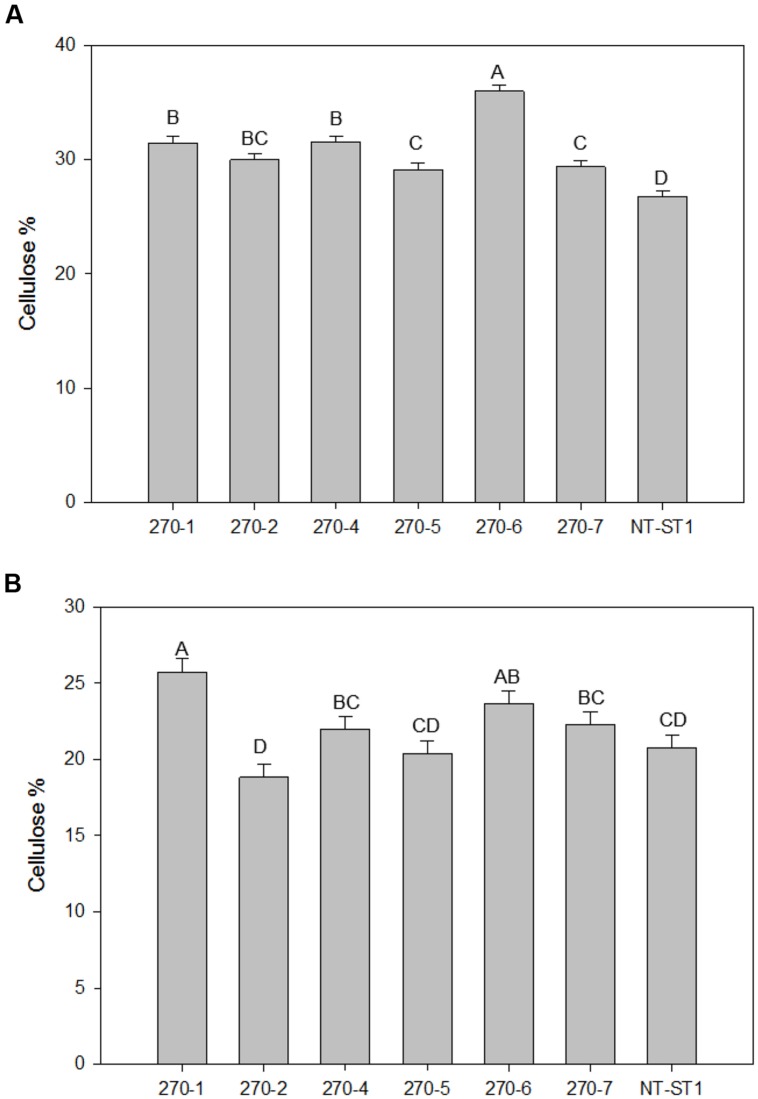
**Cellulose content in stems **(A)** and in leaves **(B)** of transgenic and non-transgenic (NT-ST1) lines as determined by Updegraff reagent.** Bars represent mean values of three biological replicates ± standard error. Bars represented by same letters are not significantly different as calculated by LSD (*p* ≤ 0.05).

In addition to a reduced level of arabinose content in the wall, most transgenic plant lines also had an increased level of xylose (up to 16%) in leaves and stems (**Figures 4C,D**). Line 270-5 was not significantly different in leaf or stem xylose content from the control line. The level of glucose in leaf wall was lower (up to 16% decreased) in most transgenic lines when compared with the control (**Figure 4E**). Lines 270-1 and 270-4 had up to 18% reduced glucose in stem, while stem glucose of the remaining lines was not significantly altered from the control (**Figure 4F**). The galactose content in stem cell wall was significantly lower (up to 60% decreased) in transgenic plant lines 270-1, 270-2, and 270-4 while lines 270-5, 270-6, and 270-7 were similar to the control (**Supplementary Table S3**). In leaves, on the other hand, the amount of galactose in the wall was slightly lower (up to 17% decreased) only in line 270-1 (**Supplementary Table S4**). The rhamnose content in leaf walls was variable among transgenic plants, with a significant increase (up to 83%) in lines 270-2, 270-4 and 270-6 (**Supplementary Table S4**) when compared with control. In stems, most transgenic lines exhibited reduced levels of wall rhamnose (up to 51%; **Supplementary Table S3**). Cell wall mannose levels in leaves and stems were similar among transgenic and control plants (**Supplementary Tables S3** and **S4**). The amount of cellulose in both stem internodes and leaves was determined as well. An increase in cellulose levels was observed in stems of all PvUAM-RNAi transgenic lines (**Figure 5A**), and in leaves, transgenic lines 270-1 and 270-6 showed a significantly higher amount of cellulose when compared to the control (**Figure 5B**). The cellulose level in leaves for lines 270-2, 270-4, 270-5, and 270-7 were not different than the control (**Figure 5B**).

Because total sugar analysis is insufficient to identify gross changes in polysaccharide structure and organization, NMR analysis of extracted arabinoxylan was performed.

### Arabinoxylan has Altered Side Chains in PvUAM-RNAi Mutants

Most of the Ara*f* residues in the cell walls of switchgrass are found in arabinoxylan side chains. To determine if the synthesis of this polymer was altered in PvUAM-RNAi transgenic plants, we analyzed arabinoxylan oligosaccharides generated by enzymatic hydrolysis of the arabinoxylan solubilized from the stem internodes and in the leaves by ^1^H-NMR. This method for example, should distinguish in principle an arabinose in the furanose form from a pyranose form and should provide linkage anomeric configurations (α- or β-form) as well as linkage positions (xylan 1–4 and any branching 1–2, 1–3, etc). In addition, NMR is an excellent method to identify and quantify resolved sugar signals in mixtures of polymers.

The ^1^H-NMR analysis of material solubilized with 1 M KOH from the stem walls and hydrolyzed with a xylanase showed clearly that the most abundant component in the sample corresponded to arabinoxylo-oligosaccharides (**Supplementary Figure S3**). The spectra contained intense signals that corresponded to the anomeric proton of α- and β-reducing Xyl. Signals for both α-Xyl and β-Xyl are detected by NMR, because the reducing end Xyl of the digested xylo-oligomers in solution undergoes opening that assumes both α and β closed-ring configurations. The NMR spectra contained two clearly resolved signals that corresponded to the anomeric proton of two types of arabinose residues. One signal (δ 5.39) was identified as terminal α-L-arabinosyl residues linked at *O*-3 to xylose in the backbone. This signals also corresponded to the terminal Ara in the disaccharide side chain α-L-Ara*f*-(1 → 2)-α-l-Ara*f*-(1 → 3)-, which has the same chemical shift and cannot be differentiated by this analysis. The other signal (δ 5.55) corresponded to Ara*f* substituted at *O*-2 with a single α-Ara*f* or a β-Xyl*p* residue. The relative amount of anomeric signal for a primary branch arabinose decorating the xylan backbone (2-α-Ara) in stems was increased in lines 270-1, 270-2, and 270-4 over control and was found to be equivalent for 270-5, 270-6, and 270-7. In stems the relative anomeric signal for terminal arabinose residues (T-α-Ara) was decreased in lines 270-1 and 270-2 compared to control while remaining lines were fairly equivalent (**Table 2**). In stems there was a marked increase in α-4-MeGlcA anomeric signal in lines 270-1, 270-2, and 270-4 while the remaining transgenic lines were equivalent to the control. The α-4-MeGlcA signal represents side-chain decoration of xylan. Line 270-1 and 270-2 have less overall arabinose branching compared to non-transgenic primarily due to the decreased in terminal arabinose (T-α-Ara) signal (**Supplementary Figure S3**). Because T-α-Ara signal is representative of H1 of arabinofuranose for both monomeric and extended branches, these data suggest stem arabinoxylan in lines 270-1 and 270-2 has relatively more extended branches (2-α-Ara) than monomer. Additionally, in these lines there appears to be increased MeGlcA decoration of the stem xylan backbone.

**Table 2:** Glycosyl side chain analysis from stems of downregulated *PvUAM1* transgenic and non-transgenic (NT-ST1) switchgrass lines.

Line	2-α-Ara (% signal)	LSD	T-α-Araf (% signal)	LSD	α-4-GlcA (% signal)	LSD
270-1	3.91 ± 0.22	A	9.27 ± 0.10	ABCD	4.27 ± 0.18	A
270-2	3.68 ± 0.20	BC	9.70 ± 0.37	D	4.46 ± 0.65	ABC
270-4	3.11 ± 0.09	CD	11.4 ± 0.12	BCD	3.32 ± 0.33	BCD
270-5	2.60 ± 0.10	CD	12.1 ± 0.16	A	2.24 ± 0.38	CD
270-6	2.35 ± 0.05	CD	11.5 ± 0.16	AB	1.97 ± 0.29	CD
270-7	2.59 ± 0.05	CD	10.6 ± 0.17	AB	2.14 ± 0.28	CD
NT-ST1	2.54 ± 0.12	D	11.2 ± 0.18	CD	2.47 ± 0.28	D


*2-α-Ara refers to the internal chains of arabinose present. T-α-Araf refers to the terminal residues of arabinose present. α-4-GlcA refers to the glucuronic acid present. Values represent relative % of ^1^H-signal of the total xylan signal. Error bars represent standard error of the mean of three stem internode replicates. Values that share the same letter are not significantly different as calculated by LSD (*p* ≤ 0.05).*

As in stems, the leaves the α-Xyl and β-Xyl were unchanged from control (**Table 3**). Leaf signal for 2-α-Ara was increased in 270-1 and 270-2 compared to control and equivalent for the other lines. Leaf signal for T-α-Ara was reduced in lines 270-1 and 270-2 compared to control. From leaves the α-4-MeGlcA signal was only markedly increased in line 270-1 (**Table 3**). These results largely indicate that leaf arabinoxylan structure in lines 270-1 and 270-2 has been altered similar to stems; relatively less overall branching of the xylan backbone with more extended branches than monomers.

**Table 3:** Glycosyl side chain analysis from leaves of downregulated *PvUAM1* transgenic and non-transgenic (NT-ST1) switchgrass lines.

Line	2-α-Ara (% signal)	LSD	T-α-Araf (% signal)	LSD	α-4-GlcA (% signal)	LSD
270-1	3.72 ± 0.22	A	9.27 ± 0.07	AB	2.87 ± 0.66	A
270-2	4.03 ± 0.07	A	9.13 ± 0.06	BC	2.04 ± 0.34	AB
270-4	2.84 ± 0.04	D	9.86 ± 0.33	D	1.95 ± 0.46	B
270-5	3.11 ± 0.20	BCD	11.5 ± 0.21	A	1.40 ± 0.33	B
270-6	3.05 ± 0.15	BC	10.7 ± 0.26	AB	1.79 ± 0.28	B
270-7	3.00 ± 0.15	CD	10.9 ± 0.03	AB	1.55 ± 0.36	B
NT-ST1	2.88 ± 0.21	CD	10.9 ± 0.28	AB	2.01 ± 0.24	AB


*2-α-Ara refers to the internal chains of arabinose present. T-α-Araf refers to the terminal residues of arabinose present. α-4-GlcA refers to the glucuronic acid present. Values represent relative % of ^1^H-signal of the total xylan signal. Error bars represent standard error of the mean of three leaf replicates. Values that share the same letter are not significantly different as calculated by LSD (*p* ≤ 0.05).*

### Saccharification of PvUAM-RNAi Lines Is Unchanged for Total Sugars

**FIGURE 6:**
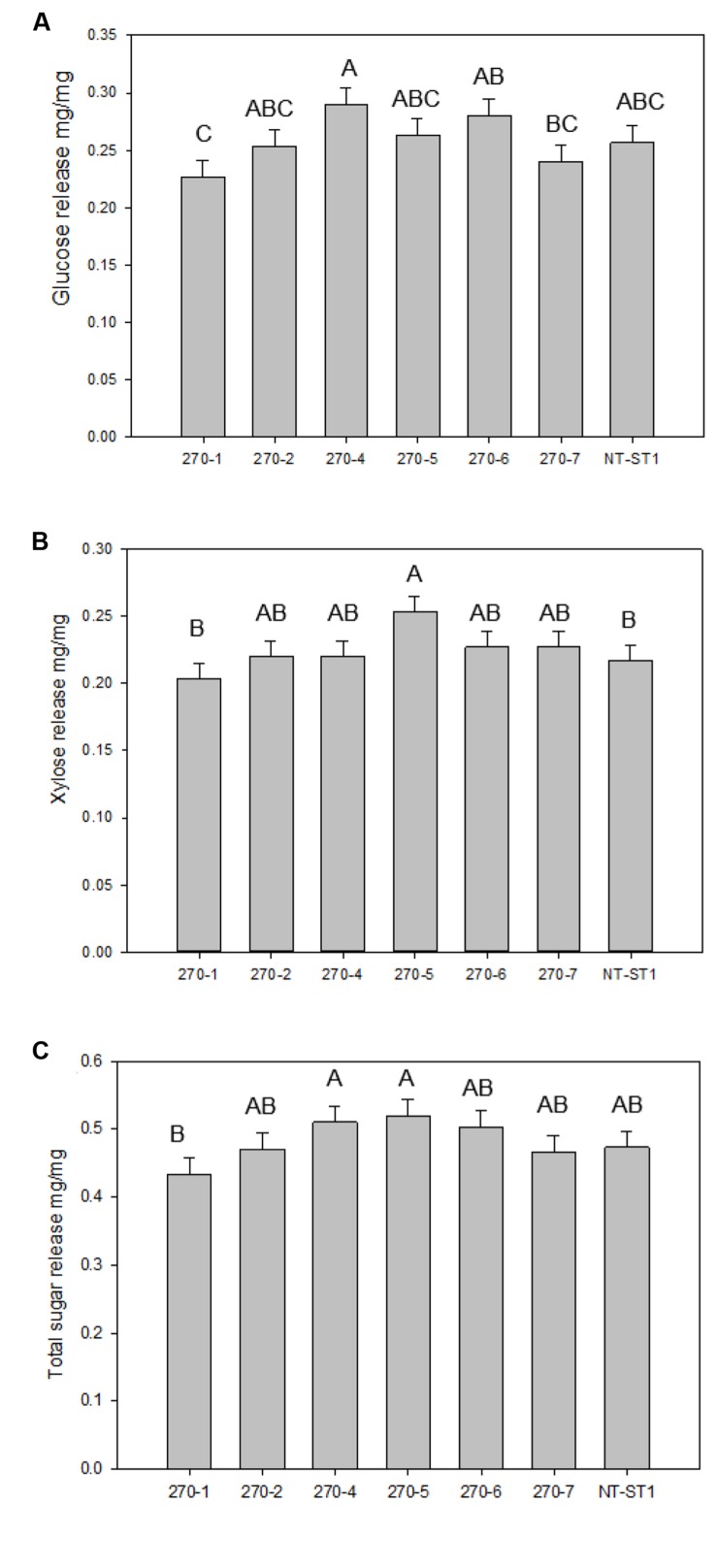
**Glucose **(A)**, xylose **(B)**, and total sugar **(C)** release from transgenic and non-transgenic (NT-ST1) whole tiller cell wall residues as determined by enzymatic hydrolysis.** Bars represent mean values of three whole plant replicates ± standard error. Bars represented by same letters are not significantly different as calculated by LSD (*p* ≤ 0.05).

PvUAM-RNAi plant cell wall sugars were analyzed for polysaccharide enzymatic release from dried R1 tillers. Enzymatic sugar release is one indicator for the level of recalcitrance of the plant cell wall against enzymatic degradation. Enzymatic glucose release was increased up to 13% for 270-4, 270-6 whereas lines 270-1, 270-2, 270-5, and 270-7 were equal to control (**Figure 6A**). Enzymatic release of xylose was significantly increased only in line 270-5 by 17% while the other transgenic lines were not different than the control (**Figure 6B**). When data for glucose and xylose release were added, there was no apparent change amongst transgenic plants and the control for total combined sugar release upon saccharification (**Figure 6C**).

### Lignin Biosynthesis: Gene Expression, Lignin Content, and Composition in Tillers

**FIGURE 7:**
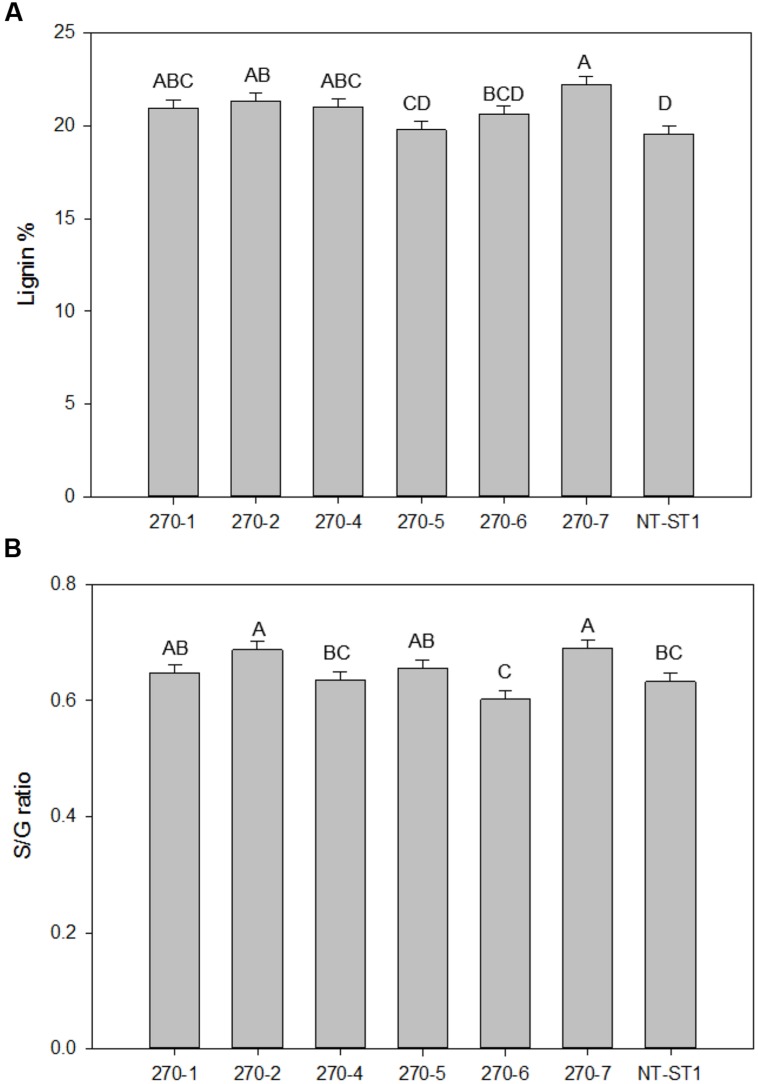
**Lignin content **(A)** and S/G ratio **(B)** of downregulated *PvUAM1* transgenic and non-transgenic (NT-ST1) whole tiller cell wall residues as determined by PyMBMS.** Bars represent mean values of three whole plant replicates ± standard error. Bars represented by same letters are not significantly different as calculated by LSD (*p* ≤ 0.05).

As high as 13% more lignin was observed in transgenic plants compared with the control (**Figure 7A**). In addition, the relative ratio of the monolignol components syringyl and guaiacyl (also known as the S/G ratio) was increased by 9 and 14%, only in transgenic plants 270-2 and 270-7, respectively (**Figure 7B**) compared with the control. Since py-MBMS lignin estimates do not include H lignin, thioacidolysis was performed on stems of control and representative transgenic lines 270-1 and 270-6. All samples had similar H lignin composition, which ranged from 4 to 6% of total lignin (**Supplementary Table S5**). Because the samples have similar low fractions of H lignin, we concluded that H monolignol effects were not biologically important. In addition, the values were within the error rate of py-MBMS total lignin analysis.

**FIGURE 8:**
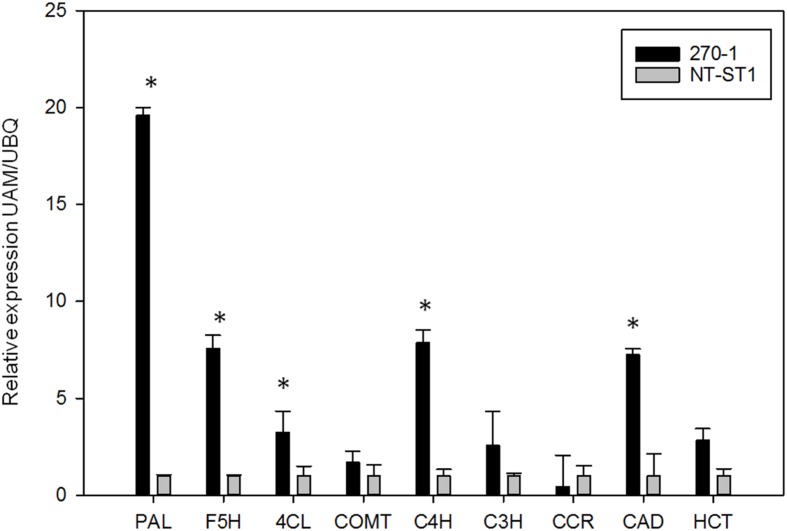
**Relative expression of lignin biosynthetic genes in transgenic (270-1) and non-transgenic (NT-ST1) stem internodes as determined by qRT-PCR.** The relative expressions were normalized to switchgrass ubiquitin 1 (*PvUbi1*; relative expression UBQ). Bars represent mean values of three replicate tiller internode ± standard error. Asterisks indicate significant differences from non-transgenic control plants at *p* ≤ 0.05 as tested by LSD. PAL, phenylalanine ammonia-lyase; F5H, ferulate 5-hydroxylase; 4CL, 4-coumarate: CoA ligase; COMT, caffeic acid 3-*O*-methyltransferase; C4H, coumaroyl shikimate 4-hydroxylase; C3H, coumaroyl shikimate 3-hydroxylase; CCR, cinnamoyl CoA reductase; CAD, cinnamyl alcohol dehydrogenase; HCT, hydroxycinnamoyl.

The finding that transgenic PvUAM-RNAi lead to increase in lignin prompted us to examine selected genes involved in lignin biosynthesis. The relative amount of gene transcript was determined by qRT-PCR. There was increased expression of *PAL. F5H. 4CL. C4H*, and *CAD* genes in PvUAM-RNAi transgenes when compared to control (**Figure 8**). Expression levels of *COMT. C3H. CCR*, and *HCT* genes were unchanged compared with the control.

## Discussion

Arabinoxylans, which comprise a relatively large portion of cell walls in grass species, likely play an important role in recalcitrance in feedstocks such as switchgrass. Arabinoxylans strengthen cell walls through cross linkages with other cell wall polysaccharides and lignin (Faik, 2010; Scheller and Ulvskov, 2010; Tan et al., 2013; Rennie and Scheller, 2014). UDP-Ara*f* has been identified as a common sugar donor for the synthesis of Ara*f*-containing side chains of the xylan backbone, which play an integral part in cross linking to other cell wall components (Anders et al., 2012). Currently the UAM class of plant proteins is the sole candidate known to convert UDP-Ara*p* to UDP-Ara*f* (Konishi et al., 2011). It has been hypothesized that decreasing the pool of available UDP-Ara*f* would, in turn, change how arabinoxylans interact with cellulose microfibrils and lignin: the reduction of numbers of cross linkages would increase the solubility of arabinoxylans (Sternemalm et al., 2008).

Hence, we propose that a reduction of *PvUAM1* would reduce arabinose side chains used for cross linkages among cell wall components, ergo, reducing recalcitrance. Our data partially supports this proposition, as the decrease in Ara*f* residues was accompanied with an increase in the total amount of lignin suggesting a compensation mechanism that has consequently resulted in unchanged cell wall recalcitrance in the transgenic lines (270-1 and 270-2).

### *PvUAM* Downregulation Affects Plant Growth

Two of the shorter plants (transgenic lines 270-1 and 270-2) had decreased expression of both *PvUAM1* and *PvUAM3*. A similar double knockdown was seen in some transgenic rice due to the close homology of the *OsUAM1* and *OsUAM3* resulting in downregulation of both homologs (Konishi et al., 2011). Transgenic switchgrass with the double knockdown showed the significant differences in cell wall-associated arabinoxylan side chains in both stems and leaves. Downregulation of only *PvUAM1* did not result in a significant change to the side chains. The transgenic switchgrass exclusively downregulated for *PvUAM1* were taller; rice *OsUAM1*-RNAi plants were shorter (Konishi et al., 2011).

Lines 270-5, 270-6, and 270-7 did not have as strong a knockdown of *PvUAM1* as events 270-1, 270-2, and 270-4. The relative attenuated knockdown might stem from effects unrelated to transgene expression, e.g., insertional effects, but for events 270-5, 270-6, and 270-7 we also did not observe significant alteration in residual Ara or arabinoxylan structure.

Based on the qRT-PCR results we hypothesize that UAM2 might not function in arabinoxylan biosynthesis, because UAM2 appears to be expressed at or over the levels of non-transgenic controls in all lines without effectively recovering arabinose content in these lines. The large overexpression of UAM3 in 270-4 is also silent in terms of glycosyl makeup. The largest effect seems instead to come from UAM1 expression, evidenced by a correlated decrease in residual Ara with PvUAM1 knockdown among all lines. While lines 270-4, 270-5, 270-6, and 270-7 have reduced residual Ara in both leaf and stem, it is not as strong as the reduction in Ara in events 270-1 and 270-2, especially in stem tissue.

Of the species we surveyed, rice UAM1 and UAM3 (*OsUAM1* and *OsUAM3*) are most homologous to *PvUAM1* and *PvUAM3* and are known to function as UDP-Ara mutases, whereas *OsUAM2*, which is homologous to *PvUAM2*, reportedly does not have mutase activity (Konishi et al., 2007). The reason *PvUAM2* expression fluctuates among events of line 270 is unknown, as is the fluctuation in *PvUAM3* expression (**Figure 2C**).

### *PvUAM* Downregulation Alters Cell Wall-Associated Sugars with no Change to Sugar Release

The phenotype we observed of decreased cell wall-associated arabinose is congruent with prior research in rice, *Arabidopsis* and *Brachypodium* (Konishi et al., 2011; Rautengarten et al., 2011; Rancour et al., 2015). In leaves, the *PvUAM1* transcript and cell wall-associated arabinose was significantly decreased in all lines except 270-5, which had the least *PvUAM1* knockdown.

As arabinose is a component of arabinoxylan, NMR was employed to deeper characterize arabinoxylan side chain structure. While a large amount of structural information can be deduced from the NMR spectra, there are regions of overlap in the anomeric signals of the xylo-oligos (Balazs et al., 2013). Specifically, the signals for monomeric α-L-Ara*f* side chains and the terminal α-L-Ara*f* in the disaccharides side chain Ara*f*-(1,2)-α-L-Ara*f*- are unresolved as are the 2-Ara*f* signals for D-Xyl*p*-(1,2)-α-L-Ara*f*-(1,3)- and α-L-Ara*f*-(1,2)-α-L-Ara*f* disaccharide side chains. However, with NMR analysis we can detect the changes in the amount of terminal and substituted Ara*f* side chains relative to the total amount of residues in the oligosaccharides. Overall, the data (**Tables 2** and **3**) suggest that the switchgrass *PvUAM*-RNAi lines with knockdown of both *PvUAM1* and *PvUAM3* have an altered arabinoxylan structure, and that the reduction of available UDP-Ara*f* causes either (1) reduced Ara*f* branching, or (2) reduced decoration of substituted side chains with a terminal Ara*f* residue (Rancour et al., 2012, 2015). Evidence from 270-1 and 270-2 stems demonstrates there is a concomitant increase in glucuronate and 4-Me-glucuronate signals, which may be due to increased substitution with GlcA to make glucuronoarabinoxylan (GAX). Arabinoxylan side chains on the other lines with only *PvUAM1* knockdown were not significantly different from the non-transgenic control.

The majority of the transgenic lines also had a slight change in the amount of residual glucose in the walls of both stems and leaves (**Figures 4E,F**). This glucose might come from small changes in xyloglucan or mixed-linkage glucan, which is known to accumulate in developing tissues (such as seed brans) and can be detected in mature stem and leaf tissues (Carpita, 1996). Of note, glucose attributed to starch was particularly increased in all transgenic lines (**Supplementary Figure S4**). In transgenic rice and *Arabidopsis* in which the relevant *UAM1* homolog had decreased expression, there was no significant change to cell wall-associated glucose (Konishi et al., 2011; Rautengarten et al., 2011). Xylose was increased in the cell walls of leaves and stems of all transgenic lines except 270-5 stem (**Figures 4C,D**). In transgenic *AtUAM1*-RNAi *Arabidopsis* xylose was increased, but in *OsUAM1*-RNAi rice in was unchanged (Konishi et al., 2011; Rautengarten et al., 2011). Transgenic *Brachypodium* with *BdUAM1* knocked down by RNAi showed an increase of xylose in the cell walls in some lines and a decrease in others (Rancour et al., 2015).

Even though an increase in cellulose (**Figures 5A,B**) content was detected, no significant change in enzymatic saccharification was found (**Figures 6A,C**). Line 270-5 had increased enzymatic xylose release, which might be attributed to the increase in available stem cell wall-associated xylose (**Figures 4D** and **6B**). Transgenic *Brachypodium* with *BdUAM1* knocked down by RNAi had slightly increased enzymatic glucose release from stems, but significantly lower release from leaves (Rancour et al., 2015). We did not analyze saccharification by organ, only whole tillers.

### *PvUAM* Downregulation Increases Lignin Content and Composition

Attenuated UAM switchgrass plants had higher lignin in tillers. Furthermore, the composition of lignin in most *PvUAM1* transgenic switchgrass shifted toward more syringyl (S) lignin units, evidenced by increased S/G ratio and no significant change in H lignin content. This shift may be explained by the concomitant increase in expression of key enzymes in the lignin biosynthesis pathway. When *BdUAM1* was knocked down in *Brachypodium* lignin was increased in the leaves, but was found to be unchanged in sheath/stem portions of tillers (Rancour et al., 2015). Lignin was not analyzed in the rice and *Arabidopsis* studies involving *UAM1* knockdown (Konishi et al., 2011; Rautengarten et al., 2011). Even though there was an increase in cell wall lignin in our study, saccharification was mostly unchanged, which contrasts to the similar study in *Brachypodium*, in which saccharification increased (**Figure 7A**; Rancour et al., 2015). For line 270-5, *PvUAM1* expression was reduced to 56% of native *PvUAM1* transcript, lignin content and composition was unchanged, while enzymatic xylose release increased. Further knockdown of *PvUAM1* expression (as seen in other lines) caused an increase in lignin production without reducing enzymatic sugar release. One might speculate that altered ferulated xylan formation affects lignification and in order to maintain proper plant growth and development compensation is made by increasing lignin content which has been reported in *Brachypodium* (Rancour et al., 2015) and now switchgrass. Ferulation is suggested to enable cross-linking of these polysaccharides to each other as well as to lignin (Molinari et al., 2013). These cross-links are believed to strengthen the cell wall and in part, contribute to the enhanced rigidity of the walls.

Examination of the converse phenotype, where lignin is downregulated shows evidence of a potential cell wall compensation mechanism as hemicellulose is increased to replace missing lignin. In the maize brown-midrib lignin mutants (*bm3*), cell wall-associated xylose content was discovered to be equivalent or higher in certain lines while arabinose, rhamnose, and xylose-substitutions decreased (Guillaumie et al., 2008). In transgenic switchgrass using RNAi to downregulate COMT lignin biosynthetic gene, an increase in hemicellulose, xylan, and arabinan was observed (Baxter et al., 2014). In both, the *bm3* maize mutants and the RNAi-COMT switchgrass lines, the disruption in lignin biosynthesis gave rise to red pigmentation attributed to an increase in cinnamaldehyde (Guillaumie et al., 2008; Fu et al., 2011). The observed darkened internodes in the PvUAM-RNAi lines may also be caused by a lignin biosynthesis metabolite shift (**Figure 3**). In-depth characterization of cell wall polysaccharides in cell wall mutants might reveal the interactions amongst cell wall biosynthesis pathways.

**FIGURE 9:**
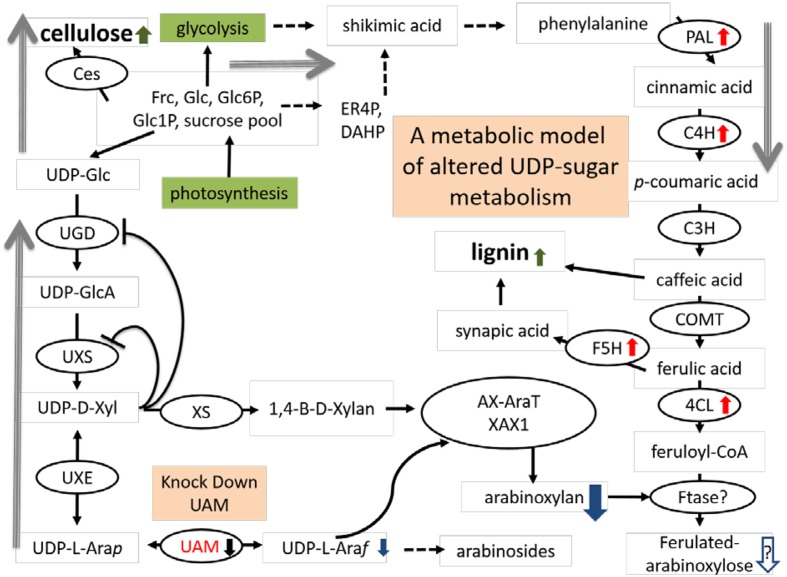
**Proposed model arabinoxylan and lignin biosynthesis pathway interactions for downregulated *PvUAM1* transgenic switchgrass.** Proposed model arabinoxylan and lignin biosynthesis pathway interactions for downregulated *PvUAM1* transgenic switchgrass. Biosynthesis proteins denoted in black ovals: Ces, cellulose synthase, UGD, uridine diphosphate (UDP)-glucose dehydrogenase; UXS, UDP-D-xylose synthase, XS, xylan synthase; UXE, UDP-xylose esterase, UAM, UDP-arabinomutase (red lettering); AX-AraT, arabinoxylan arabinosyltransferase (Porchia et al., 2002); XAX1, xylosyl-α-(1,3)-arabinosyl substitution of xylan 1 (Chiniquy et al., 2012); PAL, phenylalanine ammonia-lyase; C4H, coumaroyl shikimate 4-hydroxylase; C3H, coumaroyl shikimate 3-hydroxylase; COMT, caffeic acid 3-*O*-methyltransferase; F5H, ferulate 5-hydroxylase; 4CL, 4-coumarate: CoA ligase; Ftase?, undetermined feruloyl transferase. Red arrows indicated upregulated genes verified by qRT-PCR. Black arrow indicated downregulation of gene verified by qRT-PCR. Green arrows indicated cell wall components which have been increased. Metabolites are indicated: Frc, fructose; Glc, glucose; Glc6P, glucose-6-phosphate; Glc1P, glucose-1-phosphate; ER4P, erythrose-4-phosphate; DHAP, dihydroxyacetone phosphate; UDP-Glc, UDP-glucose; UDP-GlcA, UDP-glucuronic acid; UDP-D-Xyl, UDP-D-xylose; UDP-L-Ara*p*, UDP-L-arabinopyranose; UDP-L-Ara*f*, UDP-L-arabinofuranose. Blue arrows denote suspected reduction in metabolites resulting from downregulation of *PvUAM*. Gray arrows indicate shift in flux.

We propose a model of the interaction of hemicellulose and lignin in light of our study (**Figure 9**). In this model, an increased buildup of UDP-Xyl in UDP-Ara mutase KD-lines, leads to glucose accumulation (Glc-6-phosphate, Glc-1-P, UDP-Glc, sucrose) that is shunted toward phenylpropanoid production via the shikimic acid pathway. Shikimic acid is the precursor for phenylalanine, which is at the top of the lignin biosynthesis pathway (Whetten and Sederoff, 1995). Sucrose is converted to UDP-glucose which is either up taken by cellulose synthase (Ces) complex to form cellulose or is converted to UDP-GlcA by UDP-glucose dehydrogenase (UGD). UDP-GlcA is converted by UDP-D-xylose synthase (UXS) to UDP-D-xylose. Excess UDP-D-xylose in the Golgi stack can inhibit UGD and UXS preventing further buildup of UDP-D-xylose (Harper and Bar-Peled, 2002). UDP-D-xylose is either converted to 1,4-β-D-xylan by a xylan synthase (XS) or into the arabinose precursor UDP-L-Ara*p* (Rennie and Scheller, 2014). UDP-L-Ara*p* is converted to UDP-L-Ara*f* by UAM and then recruited into arabinose or arabinoxylan (Konishi et al., 2011; Rautengarten et al., 2011; Kotani et al., 2013; Rennie and Scheller, 2014; Rancour et al., 2015). We propose that the reduction in available UDP-L-Ara*f* caused by *PvUAM*-RNAi results in an increase of UDP-D-xylose with a corresponding reduction in arabinoxylan branching. The possible reduction in Ara*f* side chains, which are normally ferulated by an unknown feruloyl transferase, causes an increase of feruloyl-CoA. Excess ferulic and caffeic acid accumulation is shunted to lignin biosynthesis. This model is supported by the decrease in arabinose and arabinose-furanose side chains (**Figure 6**; **Tables 2** and **3**) found in the *PvUAM*-RNAi lines. The increase in lignin content and S/G ratio along with upregulation of lignin biosynthetic genes (**Figures 7** and **8**) supports the probability of increased synaptic acid levels being generated by increased F5H transcript. Further testing of hypotheses inferred by this model could be accomplished by making combinatorial knockdowns of genes that code enzymes in arabinoxylan and lignin biosynthetic pathways. Pleiotropic perturbations in gene expression and metabolic flux would be informative. Identification and characterization of the suspected feruloyl transferase would aid in discerning the complete molecular mechanism for how the cross linking between arabinoxylan and lignin occurs.

Interference with arabinofuranose metabolism has impacted cross-linking and lignin, with no evident influence on sugar release in switchgrass. The increase in lignin of *PvUAM*-RNAi plants might seem unfavorable for lignocellulosic ethanol production, however, the sugar release efficiency was not affected by the increase in lignin content. While *PvUAM*-RNAi manipulation might not solely improve bioenergy feedstocks, UAM-knockdown lines might be useful as a crossing partner with other switchgrass lines that have low lignin. Particular examples are *COMT* and *MYB4* transgenic lines modified for decreased lignin and increased sugar release efficiency, which might complement the increase in glucose, lignin, and biomass of *PvUAM*-RNAi transgenics in transgene stacks (Fu et al., 2011; Shen et al., 2012; Baxter et al., 2014, 2015). Additional switchgrass lines that might be useful to cross with UAM1 knockdown transgenics are *MYB4* and *miR156* overexpressers, of which some lines were dwarfed but yielded relatively high biofuel (Fu et al., 2012; Shen et al., 2012; Baxter et al., 2015). Additionally, any feedstock that produces inordinately high amounts of lignin might be useful for co-products, such as carbon fiber and bio-plastics (Lindsey et al., 2013; Ragauskas et al., 2014).

## Conclusion

We have identified UAM in switchgrass and the downregulated *PvUAM1* switchgrass plants have altered cell wall sugar content and side chains. Downregulation of *PvUAM1* produced a decrease in arabinose with concurrent increase in lignin content in the cell walls of transgenic switchgrass. We propose a model in which decreasing the available arabinoxylan causes an increase in lignin content due to excess metabolites not being used for arabinoxylan-lignin cross linking. Enzymatic saccharification was not negatively affected by the increase in lignin content possibly because of an increase in cellulose and mol% of xylose in walls of transgenic leaves and stems. Some transgenic *PvUAM1* plants produced increased biomass, which would be useful for commercial biomass and carbon sequestration platforms as well as a lignin feedstock.

## Author Contributions

JW drafted the manuscript, generated the majority of the transgenic plants, performed the statistical analysis, performed Southern blotting and qRT-PCR analysis, and prepared plant samples for recalcitrance analysis. JS produced the sugar profile data, collected samples for repeated recalcitrance analysis, ran NMR analysis, and contributed to manuscript drafting. MM participated in experimental design and data analysis, assisted with revisions to the manuscript and coordination of the study. J-YZ and MU assisted with cloning of the target gene. GT, SD, RS, and MD assisted with performing lignin and sugar release assays. CP and HB contributed to tissue culture and generation of transgenic plants. DM contributed to initialization of the research and early oversight of the project. MB-P and CS conceived of the study and its design and coordination. All authors contributed to text and data analysis and interpretation. All authors read and approved final version of the manuscript.

## Conflict of Interest Statement

The authors declare that the research was conducted in the absence of any commercial or financial relationships that could be construed as a potential conflict of interest.

The reviewer AR declared a shared affiliation, though no other collaboration, with several of the authors (JW, MM, CP, HB, DM, CS) to the handling Editor, who ensured that the process nevertheless met the standards of a fair and objective review.

## Footnotes

1 http://www.ebi.ac.uk/Tools/mas/muscle/

2 http://phylogeny.lirmm.fr/phylo_cgi/index.cgi

3 https://phytozome.jgi.doe.gov/pz/portal.html
