# Supplementary material for: Downregulation of a UDP-Arabinomutase Gene in Switchgrass (Panicum virgatum L.) Results in Increased Cell Wall Lignin While Reducing Arabinose-Glycans
Source: Front Plant Sci. 2016 Oct 27;7:1580. doi: 10.3389/fpls.2016.01580 (PMC5081414; doi:10.3389/fpls.2016.01580)
Supplement: Supplementary file 1 [file Data_Sheet_1.docx]

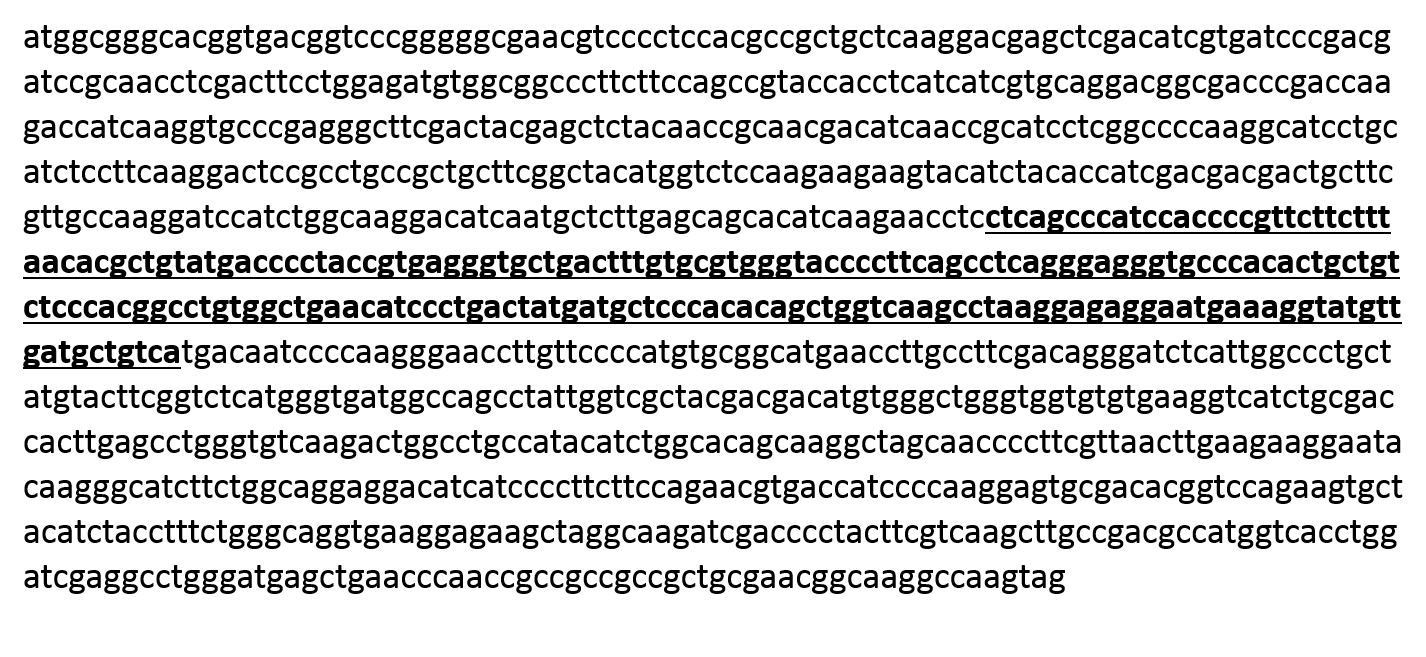


Figure S1 Full-length coding sequence of *PvUAM1*open reading frame. The 193 bp sequence selected for use in the RNAi cassette is bolded and underlined.


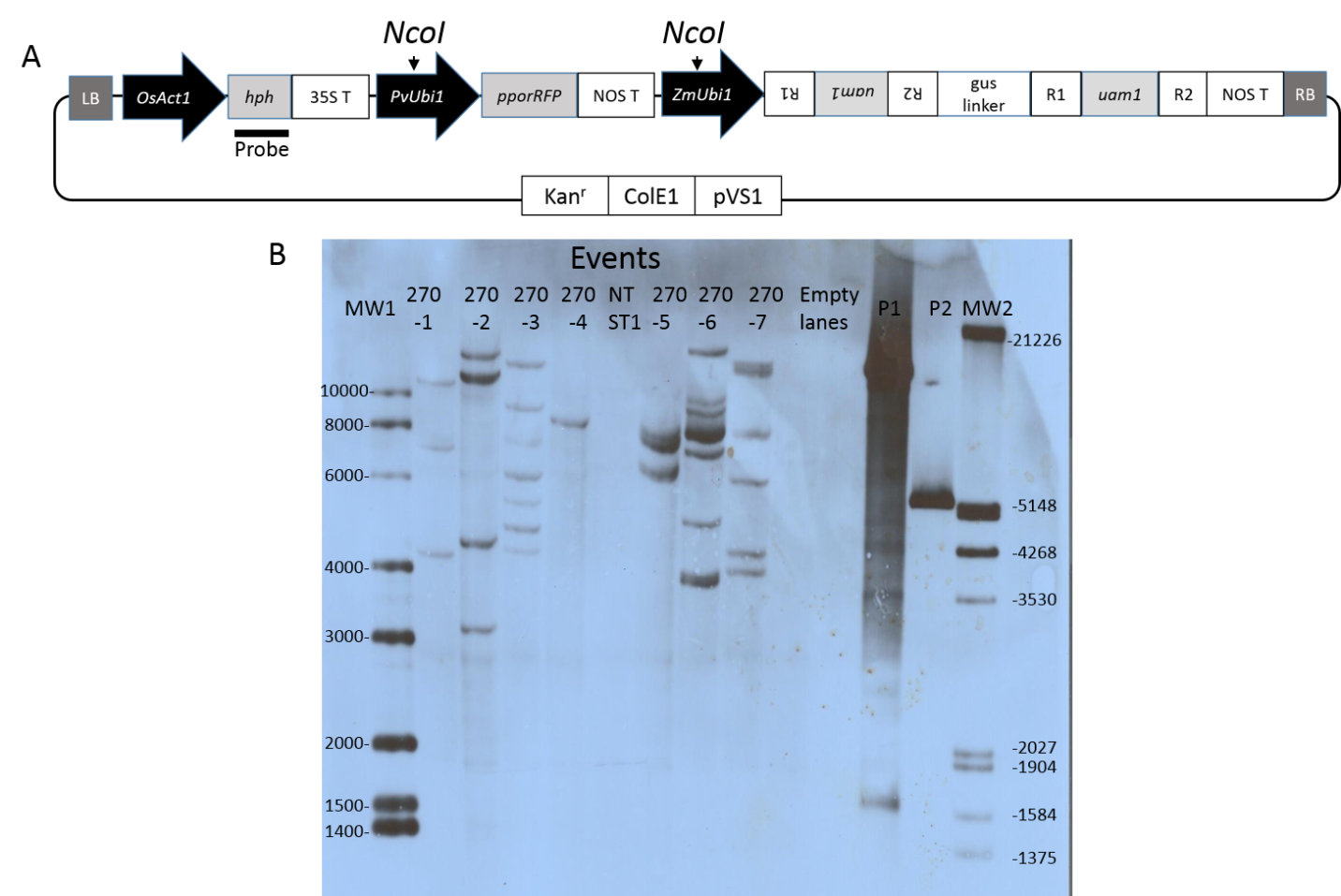


Figure S2 *PvUAM*-RNAi vector map and Southern blot for transgene insertion analysis. A) The *PvUAM*-RNAi transformation vector used the pANIC8A RNAi plant expression vector. Key features are shown including the underlined *hph* hygromycin selectable marker gene, which was used as the probe for Southern blot analysis. *NcoI* with arrows make sites where NcoI restriction enzyme cut for Southern blot analysis. LB = Left border, OsAct1 = Rice actin promoter and intron, hph = hygromycin B phosphotransferase coding region, 35S T= 35S Terminator sequence, PvUbi1 =Switchgrass ubiquitin 1 promoter and intron, pporRFP = *Porites porites* red fluorescent protein coding region, NOS T = *Agrobacterium tumefaciens nos* terminator sequence, ZmUbi1 = Maize ubiquitin 1 promoter, R1 and R2 = *attR1* and *attR2* recombinase sites 1 and 2, *uam1* = *UAM1* sequence fragment for RNAi, RB = Right border, Kan^r^ = kanamycin resistance gene for bacterial selection, ColE1 = origin of replication in *E. coli*, pVS1 = origin of replication in *A. tumefaciens,* OCS T= octopine synthase terminator sequence. B) Southern blot analysis of transgenic events and non-transgenic control (NT-ST1) switchgrass genomic DNA digested by *NcoI*. The hygromycin resistance (*hph*) gene was used as probe. P1 is an uncut pANIC8A-PvUAM1 plasmid, P2 is the linearized pcr4-hph plasmid control. MW1 is the Hi-Lo DNA marker with base pair values listed. MW2 is the DIG DNA marker III with base pair values listed.


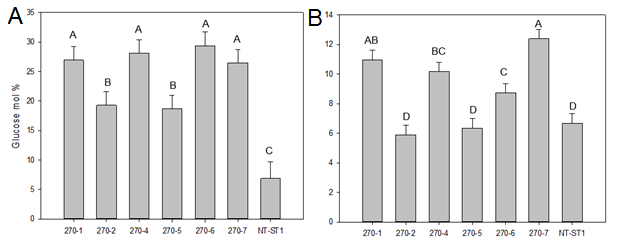


Figure S3. Glucose content in non-de-starched leaf (A) and stem (B) of transgenic and non-transgenic (NT-ST1) lines as determined by gas chromatography. Samples were normalized to internal control (inositol) with mol% representing the % of total cell wall-associated sugars measured. Bars represent mean values of three leaf or stem internode replicates ± standard error. Bars represented by same letters are not significantly different as calculated by LSD (p ≤ 0.05).


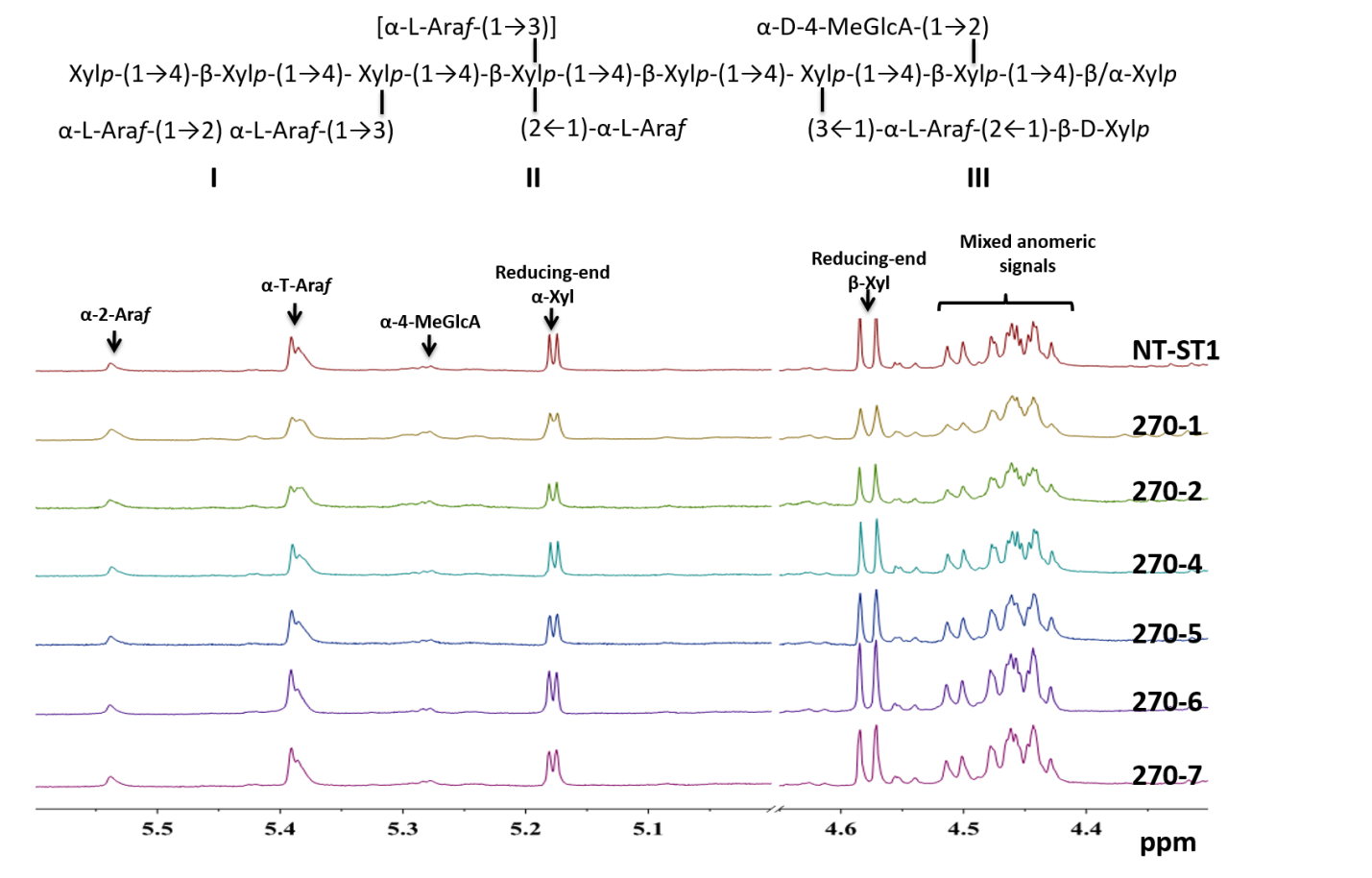


Figure S4. H^1^ NMR analysis of xylan-oligosaccharides enzymatically generated from stem arabinoxylan. Schematic of the main arabinoxylan sidechains attached at *O*-2 or *O*-3 of the β-Xyl*p*-(1→4) backbone are **I;** α-L-Ara*f*-(1→2)-α-L-Ara*f,* **II**; monomeric α-L-Ara*f*, and **III**; β-D-Xyl*p*-(1→2)-α-L-Ara*f,* **IV**; α-D-4-MeGlcA. The α-2-Ara*f* signal corresponds to H1 of α-L-Ara*f* substituted at *O*-2 (as in sidechains **I** and **III**). The α-T-Ara*f* signal corresponds to H1 of “terminal” α-L-Ara*f* residues (as in sidechains **I** and **II**).

Table S1 List of primers used for qRT-PCR analysis of *PvUAM1, PvUAM2,* and *PvUAM3*.

| PvUAM1-F | AACGTGACCATCCCCAAGGAGTG |  |
| --- | --- | --- |
| PvUAM1-R | GAAGTAGGGGTCGATCTTGCCC |  |
| PvUAM2-F | GAGAAATCAGCGGAACACAACC |  |
| PvUAM2-R | GAAAATAGCAGGCCCCAACACC |  |
| PvUAM3-F | GGCATCTTCTGGCAAGAGGAGC |  |
| PvUAM3-R | CTTCGCCCTCACCTGCTTGGCC |  |

Table S2 List of primers used for qRT-PCR analysis of lignin biosynthetic genes.

| C4H1_1534F | GGGCAGTTCAGCAACCAGAT | Shen, H. et al., 2012 |
| --- | --- | --- |
| C4H1_1611R | CGCGTTTCCGGGACTCTAG | Shen, H. et al., 2012 |
| PvCOMT_F461 | CAACCGCGTGTTCAACGA | Shen, H. et al., 2012 |
| PvCOMT_R534 | CGGTGTAGAACTCGAGCAGCTT | Shen, H. et al., 2012 |
| 4CL1_1179_F | CGAGCAGATCATGAAAGGTTACC | Shen, H. et al., 2012 |
| 4CL1_1251_R | CAGCCAGCCGTCCTTGTC | Shen, H. et al., 2012 |
| PvCCR1. 112_F | GCGTCGTGGCTCGTCAA | Shen, H. et al., 2012 |
| PvCCR1. 187_R | TCGGGTCATCTGGGTTCCT | Shen, H. et al., 2012 |
| PvCAD_F116 | TCACATCAAGCATCCACCATCT | Shen, H. et al., 2012 |
| PvCAD_R184 | GTTCTCGTGTCCGAGGTGTGT | Shen, H. et al., 2012 |
| HCT_973_F | GCAGAAGGAGCAGCAGTCATC | Shen, H. et al., 2012 |
| HCT_1035_R | CGAGCGGCAATAGTCGTTGT | Shen, H. et al., 2012 |
| PAL_F1 | CATATAGTGTGCGTGCGTGTGT | Wuddineh, W. et al., 2014 |
| PAL_R1 | CTGGCCCGCCAATCG | Wuddineh, W. et al., 2014 |
| C3H_F1 | CGTGAACAATGGGATCAGGATAG | Wuddineh, W. et al., 2014 |
| C3H_R1 | GCGGACACAACCATCTCAAATAC | Wuddineh, W. et al., 2014 |
| F5H_F1 | CCCCGTGCACTGACGATCTAT | Wuddineh, W. et al., 2014 |
| F5H_R1 | CCAAGCCAAGGGAAAACACAGTTA | Wuddineh, W. et al., 2014 |
| F_PvUBIQUITIN | CAGCGAGGGCTCAATAATTCCA | Xu, B. et al., 2011 |
| R_PvUBIQUITIN | TCTGGCGGACTACAATATCCA | Xu, B. et al., 2011 |

Table S3 Galactose, rhamnose, and mannose content in stems of transgenic and non-transgenic (NT-ST1) events as determined by gas chromatography. Samples were normalized to the non-transgenic control with mol% representing the % of total cell wall-associated sugars measured. Values represent mean of three stem internode replicates ± standard error. Values with same letters are not significantly different as calculated by LSD (*p* ≤ 0.05).

| Event | Galactose (mol%) | LSD | Rhamnose (mol%) | LSD | Mannose (mol%) | LSD |
| --- | --- | --- | --- | --- | --- | --- |
| 270-1 | 1.70 ± 0.03 | D | 0.41 ± 0.02 | D | 0.48 ± 0.04 | A |
| 270-2 | 2.96 ± 0.18 | C | 0.53 ± 0.01 | CD | 0.58 ± 0.06 | A |
| 270-4 | 2.96 ± 0.13 | C | 0.55 ± 0.05 | CD | 0.65 ± 0.10 | A |
| 270-5 | 4.38 ± 0.44 | A | 0.87 ± 0.07 | A | 0.77 ± 0.11 | A |
| 270-6 | 3.52 ± 0.37 | BC | 0.69 ± 0.05 | BC | 0.75 ± 0.13 | A |
| 270-7 | 3.92 ± 0.23 | AB | 0.75 ± 0.09 | AB | 0.76 ± 0.12 | A |
| NT-ST1 | 4.20 ± 0.30 | AB | 0.84 ± 0.06 | AB | 0.79 ± 0.13 | A |

Table S4 Galactose, rhamnose, and mannose content in leaves of transgenic and non-transgenic (NT-ST1) lines as determined by gas chromatography. Samples were normalized to the non-transgenic control with mol% representing the % of total cell wall-associated sugars measured. Values represent mean of three leaf replicates ± standard error. Values with same letter are not significantly different as calculated by LSD (*p* ≤ 0.05).

| Event | Galactose (mol%) | LSD | Rhamnose (mol%) | LSD | Mannose (mol%) | LSD |
| --- | --- | --- | --- | --- | --- | --- |
| 270-1 | 2.93 ± 0.17 | D | 0.52 ± 0.09 | C | 0.58 ± 0.08 | BC |
| 270-2 | 3.96 ± 0.39 | AB | 0.80 ± 0.03 | AB | 0.68 ± 0.05 | AB |
| 270-4 | 4.25 ± 0.28 | A | 0.95 ±0.115 | A | 0.66 ±0.05 | ABC |
| 270-5 | 3.15 ± 0.07 | CD | 0.68 ± 0.01 | BC | 0.43 ± 0.03 | C |
| 270-6 | 3.76 ± 0.10 | ABC | 0.96 ± 0.01 | A | 0.86 ± 0.14 | A |
| 270-7 | 2.98 ± 0.18 | D | 0.60 ± 0.02 | C | 0.47 ± 0.03 | BC |
| NT-ST1 | 3.53 ± 0.01 | BCD | 0.65 ± 0.07 | BC | 0.61 ± 0.08 | BC |

Table S5 H lignin content in 270-1, 270-6 and NT-ST1 as determined by thioacidolysis. Values represent mean of three R1 whole tiller replicates ± standard error.

|  | **H µmol/g biomass** | **% H** |
| --- | --- | --- |
| 270-1 | 6.82 ± 0.51 | 4.54 ± 0.60 |
| 270-6 | 6.34 ± 0.34 | 4.68 ± 0.24 |
| NT-ST1 | 6.82 ± 0.60 | 5.42 ± 0.38 |
